# Colorectal Cancer With High Tumor Mutational Burden and Mesenchymal-Epithelial Transition (MET) Amplification With Hyperprogressive Disease After Pembrolizumab Treatment

**DOI:** 10.7759/cureus.85954

**Published:** 2025-06-13

**Authors:** Satoru Nakajima, Akinori Sasaki, Risa Okamoto

**Affiliations:** 1 Gastroenterology, Tokyo Bay Urayasu Ichikawa Medical Center, Urayasu, JPN

**Keywords:** colorectal cancer, hyperprogressive disease, met amplification, pembrolizumab, tumor mutational burden-high

## Abstract

Pembrolizumab, an immune checkpoint inhibitor, has shown efficacy in tumor mutational burden-high (TMB-H) solid tumors and has been approved for the treatment of these diseases. Following immune checkpoint inhibitor administration, rapid tumor progression, known as hyperprogressive disease (HPD), has been observed. This report presents the case of a 60-year-old woman diagnosed with mesenchymal-epithelial transition (MET) amplification and TMB-H colorectal cancer. The patient was initially administered chemotherapy for MET amplification in a clinical trial but was considered refractory following one treatment cycle. Subsequently, she was treated with pembrolizumab for the TMB-H solid tumor. However, she developed HPD one month after starting pembrolizumab treatment and later died in the hospital. To the best of our knowledge, this is the first report of HPD in a patient with colorectal cancer harboring both MET amplification and TMB-H. It suggests that MET amplification may be involved in HPD development. These findings underscore the need for vigilance regarding HPD risk when selecting immune checkpoint inhibitor candidates and highlight the importance of future research, such as exploring MET-targeted combination strategies, in the optimization of treatment outcomes.

## Introduction

Colorectal cancer (CRC) is the third most common cancer and the second leading cause of cancer-related deaths worldwide [1]. Chemotherapy regimens, including oxaliplatin, irinotecan, and fluoropyrimidine, remain the standard treatment for unresectable CRC [2, 3]. 

Recently, comprehensive genomic profiling (CGP) has been approved as a guiding tool for precision-centered oncological treatment of solid tumors, including CRC, and is now being used in clinical practice. In some cases, molecular targeted therapies can be selected when target gene mutations are identified using CGP. The efficacy of immune checkpoint inhibitors (ICI) in CRC has not been proven. However, pembrolizumab, an anti-programmed death-1 (PD-1) antibody, is effective and has been approved for patients with CRC harboring high microsatellite instability (MSI-H) or high tumor mutational burden (TMB-H), as detected by CGP [4, 5]. In contrast, ICIs have been associated with rapid immunosuppression, a condition known as hyperprogressive disease (HPD), in various cancer types [6].

Although the efficacy of ICIs in MSI-H and TMB-H CRC has been proven, HPD development has also been observed [6]. HPD is defined as a rapid tumor growth of ≥50% within two months of ICI administration. This condition occurs in approximately 10-20% of various cancer cases [7]. Additionally, mesenchymal-epithelial transition (MET) amplification has been implicated in promoting tumor proliferation and immune evasion, potentially conferring resistance to ICIs and contributing to HPD [8]. Nevertheless, few studies have reported HPD in patients with TMB-H CRC receiving pembrolizumab.

This report describes the case of a patient with CRC harboring both TMB-H and MET amplifications who developed HPD in response to pembrolizumab therapy. The potential for HPD occurrence in such cases and its clinical implications are also discussed.

## Case presentation

**Figure 1:**
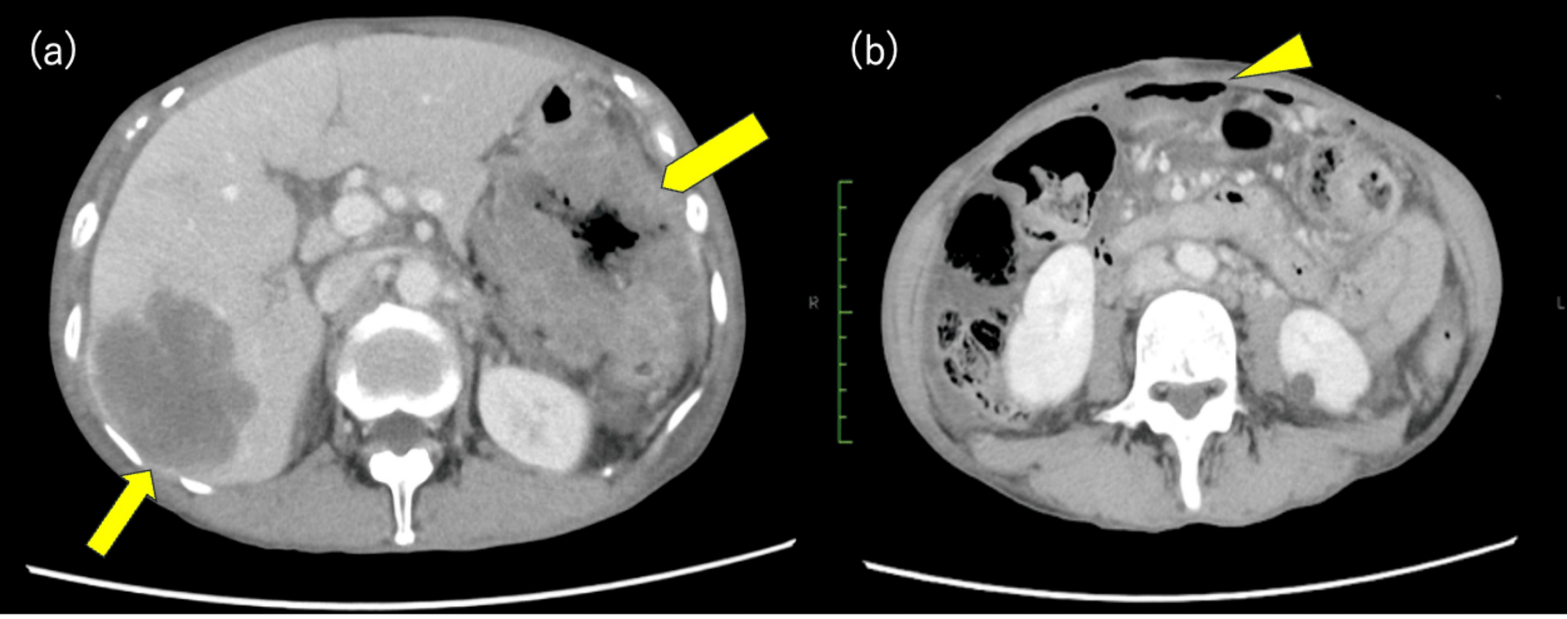
Abdominal computed tomography (CT) findings at presentation. (a) Multiple hepatic metastases (arrow) and a primary colorectal tumor with significant wall thickening (arrowhead); (b) free air in the abdominal cavity, indicative of intestinal perforation (triangle marker).

A 60-year-old woman presenting with weight loss and epigastric pain was referred to our hospital. She had a medical history of appendectomy. Computed tomography (CT) revealed a thickening of the transverse colon, multiple enlarged lymph nodes, and multiple liver masses (Figure 1a). Furthermore, CT showed the presence of free air and ascites within the abdominal cavity, leading to a diagnosis of intestinal perforation due to colon cancer (Figure 1b). Subsequently, the patient underwent emergency surgery for a perforated transverse colon. Primary tumor resection and stoma creation were performed with intraperitoneal drain placement.

Pathological examination of the surgical specimen revealed poorly differentiated adenocarcinoma. The patient was subsequently diagnosed with stage IV colon cancer (T4bN3M1, according to the TNM classification, 9th edition).

**Figure 2:**
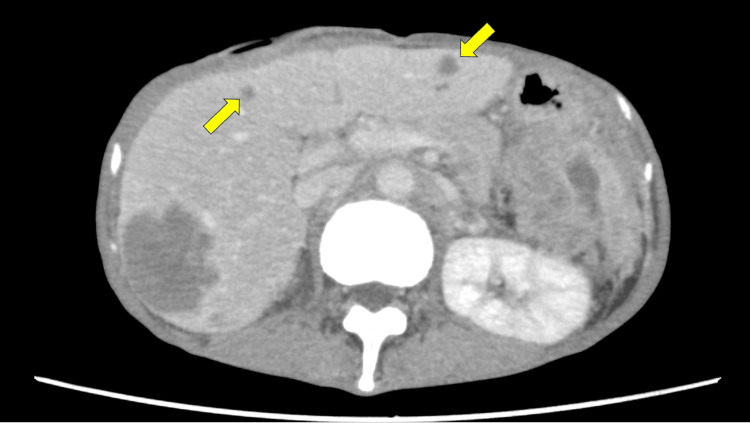
Abdominal CT after FOLFOX6 plus bevacizumab therapy. The scan shows the appearance of new liver metastases (arrow).

The patient’s general condition gradually improved after surgery and was considered eligible for chemotherapy. The patient received first-line chemotherapy with a modified FOLFOX6 plus bevacizumab regimen every two weeks: oxaliplatin 85 mg/m^2^, leucovorin 400 mg/m^2^, 5-FU bolus 400 mg/m^2^, continuous infusion of 5-FU 2400 mg/m^2^ over 46 h, and bevacizumab 5 mg/kg. However, after three cycles of treatment, an abdominal CT scan showed the appearance of new liver metastases, which led to the patient being considered refractory to first-line chemotherapy (Figure 2). Consequently, the patient was prescribed FOLFIRI plus bevacizumab as second-line chemotherapy: irinotecan 150 mg/m^2^, leucovorin 400 mg/m^2^, 5-FU bolus 400 mg/m^2^, continuous infusion of 5-FU 2400 mg/m^2^ over 46 h, and bevacizumab 5 mg/kg.

**Figure 3:**
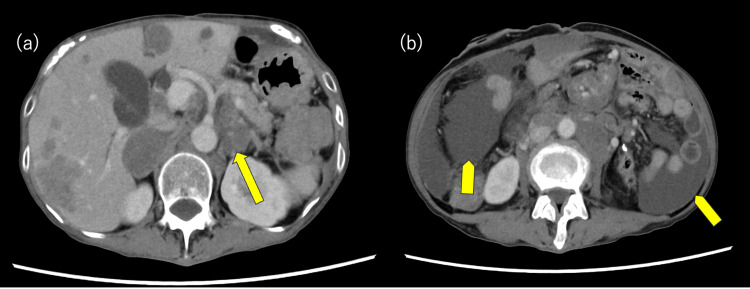
Abdominal CT scan performed one month after ABBV-400 administration. (a) An increase in lymph node enlargement (arrow); (b) the appearance of ascites (arrowhead).

While receiving second-line chemotherapy, multigene panel testing using Foundation One CDx (Foundation Medicine, Cambridge, MA) was performed on the primary tumor tissue. This testing revealed MET amplification and TMB-H (TMB: 10.8 Muts/Mb) as actionable genomic alterations. Based on the results of the multigene panel-based CGP, a therapeutic strategy was developed by an intra-institutional molecular tumor board (called an expert panel), and treatment with a c-Met Antibody-Drug Conjugate (ADC) was recommended. The patient started ABBV-400 (c-Met ADC) by participating in the clinical trial according to the expert panel’s recommendation. However, a CT scan performed one month after ABBV-400 administration revealed progression of lymph node metastases and ascites. Therefore, the patient was considered refractory to this treatment (Figure 3).

The patient’s general condition deteriorated to an Eastern Cooperative Oncology Group (ECOG) performance status (PS) of 2 because of tumor growth [9]; however, she wished to continue chemotherapy. Subsequently, pembrolizumab (200 mg/body), an anti-PD-1 antibody, was administered to target the TMB-H characteristics. CGP confirmed that the tumor was TMB-H, and based on the results of the KEYNOTE-158 trial [5], pembrolizumab is reimbursed for TMB-H solid tumors, including CRC. Hence, this therapy was selected in accordance with those guidelines.

**Figure 4:**
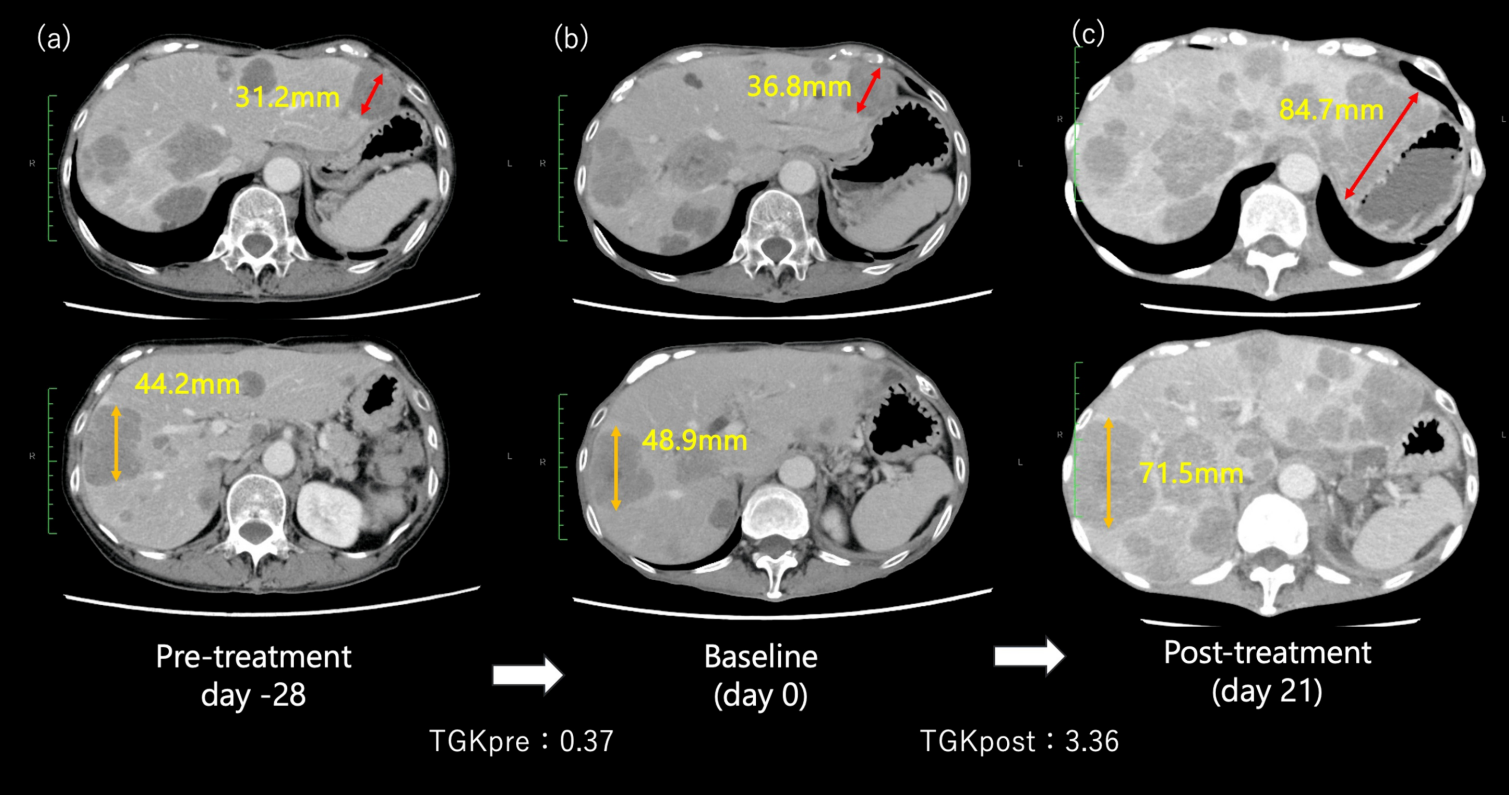
Assessment of hyperprogressive disease (HPD) using tumor growth kinetics (TGK). To determine hyperprogressive disease (HPD), we calculated the tumor growth kinetics (TGK) during the periods before pembrolizumab administration (a→b) and after its administration (b→c). Based on the results, we concluded that these findings met the criteria for HPD.

Although no significant adverse events were observed after the initiation of treatment, the patient presented to the emergency department three weeks later with severe fatigue and loss of appetite. CT confirmed rapid progression of liver and lymph node metastases, with the total tumor size increasing from 85.7 mm to 156.2 mm, meeting the criteria for HPD (Figure 4). To further substantiate this rapid progression, the tumor growth kinetics (TGK) were calculated pre- and post-pembrolizumab administration [10]. Over the 28 days prior to pembrolizumab initiation, the TGK (TGKpre) was 0.37, whereas during the following 21 days, the TGK (TGKpost) markedly rose to 3.36, suggesting significant acceleration in tumor growth, consistent with HPD. Although this is a single-case analysis, the marked elevation in TGK indicates that pembrolizumab treatment may have been associated with rapid tumor progression in this patient. Serum lactate dehydrogenase (LDH) levels and neutrophil-lymphocyte ratio (NLR) also showed elevated values prior to and during treatment (LDH from 2038 IU/L to 5644 IU/L, NLR from 22 to 88), suggesting an increasingly immunosuppressive or pro-tumor environment consistent with HPD risk factors [11]. Chemotherapy was discontinued because the patient’s condition did not improve. The patient received palliative care and died in the hospital 14 days later.

## Discussion

This study describes the development of HPD following pembrolizumab treatment in a patient with CRC harboring TMB-H and MET amplification. TMB is a measure of the total number of somatic coding mutations in tumor cells, expressed as the number of gene mutations per million bases (mut/Mb). A TMB of ≥10 mut/Mb is defined as a TMB-H [12]. In a phase II KEYNOTE-158 study, administering pembrolizumab to patients with TMB-H solid tumors, regardless of cancer type, demonstrated a response rate of 30% and a median progression-free survival rate of 2.1 months [5]. Consequently, pembrolizumab has been approved for the treatment of TMB-H solid tumors in clinical practice. Previous studies have reported rapid tumor growth and HPD after ICI administration [9,13,14]. However, few studies have reported HPD in patients with solid tumors and TMB-H receiving ICI treatment, as in this case.

Although the exact mechanism of HPD in this case is unclear, three potential causes of HPD in patients with TMB-H and MET amplification are considered. First, liver metastases with a large tumor burden may have contributed to HPD, as reported by previous studies [6, 13]. Regulatory T cells (Tregs) also contribute to the tumor microenvironment (TME) during liver metastasis. Tregs are immunosuppressive cells that contribute to the occurrence of HPD [15]. Moreover, the expression of Tregs in the TME is higher in liver metastases than in primary lesions or other metastatic organs. Lactic acid levels increase with the acceleration of glycolysis in liver metastases. Among the T cells, only eTregs can be activated in the TME with high lactic acid concentrations; therefore, eTregs are highly expressed in liver metastases. This expression may be related to HPD onset following ICI administration [15]. In this case, immunological assessments, such as Treg profiling, were not conducted. However, the accumulation of similar cases and further evaluation could help elucidate the mechanisms underlying HPD in TMB-H tumors despite their high mutational burden.

Second, poor PS at ICI administration may be involved in rapid tumor progression. Generally, the effect of ICI is poorer in patients with poor PS than in those with good PS [16]. Additionally, a meta-analysis reported a relationship between poor PS and HPD [6]. Poor PS has been correlated with high NLR and LDH levels, and both have been reported as risk factors for HPD [6, 17]. Our patient had a poor PS and elevated NLR and LDH levels, suggesting that these factors were associated with the onset of HPD.

Finally, MET amplification may be associated with HPD development. The c-MET proto-oncogene is located on human chromosome 7q21-31 and promotes cell proliferation, migration, and angiogenesis by activating the RAS and PI3 kinase pathways [18]. MET amplification causes an abnormal increase in the number of copies of the c-MET proto-oncogene, leading to the uncontrolled growth of cancer cells and tumor formation. Moreover, patients with MET amplification respond poorly to ICIs, such as anti-PD-1 antibodies [8]. A previous study suggested that the activation of the MET pathway induces an increase in immunosuppressive macrophages and suppresses antitumor immunity in the TME [19]. Additionally, a study identified MET amplification as a risk factor for HPD development in patients with non-small cell lung cancer [20]. In this case, HPD development may be associated with a combination of these risk factors. The ICIs, including pembrolizumab, have fewer side effects than cytotoxic chemotherapy and can be administered to patients with poor PS. However, it is crucial to understand that there is a risk of developing immune-mediated adverse events and HPD due to ICI administration.

This report describes a single case of CRC harboring both TMB-H and MET amplification that developed HPD after pembrolizumab treatment. Consequently, broadly generalizing the findings of this single case is challenging. Moreover, the absence of a control group or comparator makes it difficult to rigorously establish a causal relationship between pembrolizumab and rapid disease progression. Furthermore, we could not perform immunological profiling; therefore, we could not directly assess the specific immunological mechanism whereby MET amplification leads to HPD development. Further accumulation of similar cases and the implementation of prospective studies are needed to elucidate the mechanisms involved and develop more effective treatment strategies.

## Conclusions

To the best of our knowledge, this is the first report of HPD following pembrolizumab treatment in a patient with CRC harboring TMB-H and MET amplification. The approval of pembrolizumab for patients with CRC and TMB-H has increased treatment options. However, the possibility of developing HPD worsens the prognosis. Caution is necessary when administering ICI to patients with risk factors for HPD, as in this case. To develop HPD prevention strategies, several future clinical trials must be conducted using combination treatments of MET inhibitors and ICIs for MET amplification to accumulate more cases and perform further analyses of these phenomena.

## References

[1] Bray F, Laversanne M, Sung H, Ferlay J, Siegel RL, Soerjomataram I, Jemal A (2024). Global cancer statistics 2022: GLOBOCAN estimates of incidence and mortality worldwide for 36 cancers in 185 countries. CA Cancer J Clin.

[2] Van Cutsem E, Cervantes A, Adam R (2016). ESMO consensus guidelines for the management of patients with metastatic colorectal cancer. Ann Oncol.

[3] Yoshino T, Arnold D, Taniguchi H (2018). Pan-Asian adapted ESMO consensus guidelines for the management of patients with metastatic colorectal cancer: a JSMO-ESMO initiative endorsed by CSCO, KACO, MOS, SSO and TOS. Ann Oncol.

[4] Marabelle A, Le DT, Ascierto PA (2020). Efficacy of pembrolizumab in patients with noncolorectal high microsatellite instability/mismatch repair-deficient cancer: results from the phase II KEYNOTE-158 study. J Clin Oncol.

[5] Marabelle A, Fakih M, Lopez J (2020). Association of tumour mutational burden with outcomes in patients with advanced solid tumours treated with pembrolizumab: prospective biomarker analysis of the multicohort, open-label, phase 2 KEYNOTE-158 study. Lancet Oncol.

[6] Zhao Z, Bian J, Zhang J, Zhang T, Lu X (2022). Hyperprogressive disease in patients suffering from solid malignancies treated by immune checkpoint inhibitors: A systematic review and meta-analysis. Front Oncol.

[7] Wang X, Wang F, Zhong M, Yarden Y, Fu L (2020). The biomarkers of hyperprogressive disease in PD-1/PD-L1 blockage therapy. Mol Cancer.

[8] Zhang Y, Yang Q, Zeng X (2021). MET amplification attenuates lung tumor response to immunotherapy by inhibiting STING. Cancer Discov.

[9] Oken MM, Creech RH, Tormey DC, Horton J, Davis TE, McFadden ET, Carbone PP (1982). Toxicity and response criteria of the Eastern Cooperative Oncology Group. Am J Clin Oncol.

[10] Champiat S, Dercle L, Ammari S (2017). Hyperprogressive disease is a new pattern of progression in cancer patients treated by anti-PD-1/PD-L1. Clin Cancer Res.

[11] Ferrara R, Mezquita L, Texier M (2018). Hyperprogressive disease in patients with advanced non-small cell lung cancer treated with PD-1/PD-L1 inhibitors or with single-agent chemotherapy. JAMA Oncol.

[12] Alexandrov LB, Nik-Zainal S, Wedge DC (2013). Signatures of mutational processes in human cancer. Nature.

[13] Sasaki A, Nakamura Y, Mishima S (2019). Predictive factors for hyperprogressive disease during nivolumab as anti-PD1 treatment in patients with advanced gastric cancer. Gastric Cancer.

[14] Saâda-Bouzid E, Defaucheux C, Karabajakian A (2017). Hyperprogression during anti-PD-1/PD-L1 therapy in patients with recurrent and/or metastatic head and neck squamous cell carcinoma. Ann Oncol.

[15] Kumagai S, Koyama S, Itahashi K (2022). Lactic acid promotes PD-1 expression in regulatory T cells in highly glycolytic tumor microenvironments. Cancer Cell.

[16] Lin SY, Yang CY, Liao BC (2018). Tumor PD-L1 expression and clinical outcomes in advanced-stage non-small cell lung cancer patients treated with nivolumab or pembrolizumab: real-world data in Taiwan. J Cancer.

[17] Rapoport BL, Theron AJ, Vorobiof DA (2020). Prognostic significance of the neutrophil/lymphocyte ratio in patients undergoing treatment with nivolumab for recurrent non-small-cell lung cancer. Lung Cancer Manag.

[18] Organ SL, Tsao MS (2011). An overview of the c-MET signaling pathway. Ther Adv Med Oncol.

[19] Tumeh PC, Harview CL, Yearley JH (2014). PD-1 blockade induces responses by inhibiting adaptive immune resistance. Nature.

[20] Sun D, Tao J, Yan W (2022). Optimal treatments for NSCLC patients harboring primary or acquired MET amplification. Technol Cancer Res Treat.

